# Supplementary material for: Asynchronous magnetic resonance elastography: Shear wave speed reconstruction using noise correlation of incoherent waves
Source: Magn Reson Med. 2022 Oct 27;89(3):990–1001. doi: 10.1002/mrm.29502 (PMC9792433; doi:10.1002/mrm.29502)
Supplement: Supplementary file 1 — DATA S1 MATLAB code used to generate the numerical simulations of Figure 1 [file MRM-89-990-s002.zip › k-Wave/helpfiles/example_na_modelling_absorption.html]

Modelling Power Law Absorption Example (k-Wave)


# Modelling Power Law Absorption Example

This example describes the characteristics of the absorption and dispersion encapsulated by the k-Wave simulation functions.

- Open the file in the MATLAB Editor
- Run the file in MATLAB

For a more detailed discussion of the absorption model used in k-Wave, see Treeby, B. E. and Cox, B. T., "Modeling power law absorption and dispersion for acoustic propagation using the fractional Laplacian," *J. Acoust. Soc. Am.*, vol. 127, no. 5, pp. 2741-2748, 2010.

## Contents

- Absorption and dispersion characteristics
- Numerical errors

## Absorption and dispersion characteristics

The acoustic absorption within k-Wave is modelled using two phenomenological loss terms. These separately account for absorption that follows a frequency power law in addition to the associated dispersion (dependence of the sound speed on frequency) required by the Kramers-Kronig relations. Under the smallness approximation that the total absorption is much less than the acoustic wavenumber (a condition satisfied for almost all cases of interest in biomedical ultrasound and photoacoustics), these terms allow a wide range of power law absorption characteristics to be accurately modelled. This particular form of absorption is of practical relevance in a number of acoustic applications. For example, the absorption in soft biological tissue over diagnostic ultrasound frequencies has been experimentally shown to follow a frequency power law in which the exponent is between 1 and 2. Similarly, the absorption in marine sediments follows a power law where the exponent is close to 1.

Within the k-Wave simulation functions, the power law absorption is specified by two parameters, `medium.alpha_coeff` and `medium.alpha_power`. These correspond to the power law pre-factor and exponent, respectively, where the pre-factor is given in units of dB / (MHz^y cm). To illustrate the characteristics of the absorption model, the encapsulated absorption and dispersion for a range of different power absorption parameters are shown below (open circles). These are extracted from the amplitude and phase spectrums of the signals recorded at two different sensor positions in an absorbing medium in 1D using `kspaceSecondOrder` (set `example_number = 1` within the example m-file). The theoretical curves for absorption and dispersion (calculated using `powerLawKramersKronig`) are also shown for comparison (solid line). There is a close agreement between the theoretical and numerical results which demonstrates that the desired absorption characteristics have been correctly modelled.


## Numerical errors

If the same example is calculated using `kspaceFirstOrder1D` (set `example_number = 2` within the example m-file), at higher frequencies there is a small deviation from the theoretical absorption curve and a noticeable deviation from the theoretical dispersion curve.
This is due to the dependence of the loss terms on acoustical quantities in addition to their temporal gradients which are calculated at different stages within the model.
This introduces small phase errors which are accentuated as the absolute absorption level is increased.


These errors can be minimised by reducing the time step used in the simulation (set `example_number = 3` within the example m-file).
In the limit, the encapsulated absorption and dispersion properties approach those modelled by `kspaceSecondOrder`.
